# Going without a Religion

**Published:** 1885-05

**Authors:** 


### Going Without a Religion.

Our minister to England, Mr. James Russell Lowell, some time since attended a meeting, held in London, to do honor to the poet Browning. Several of the speakers, during the delivery of addresses, took occasion to refer in a sneering manner to certain forms of Christian faith, claiming that they could get along in life without any religion,—belittleing its influences upon humanity, and altogether refusing to acknowledge its claims to subdue the sinful part of man. Mr. Lowell replied to these remarks as follows: He said, "I do not think it safe—I am formulating no creed of my own; I have always been a liberal thinker, and have, therefore, allowed others, who differed with me, to think also as they liked—but at the same time I fear that when we indulge ourselves in the amusement of going without a religion, we are not, perhaps, aware how much we are sustained at present by an enormous mass all about us of religious feeling and religious conviction, so that, whatever it may be safe for us to think, for us who have had great advantages, and have been brought up in such a way that a certain moral direction has been given to our character, I do not know what would become of the less favored classes of mankind if *they* undertook to play the same game. I wished only to enter the protest of one in whose veins runs the blood of Calvanistic ancestors, against the way in which Calvanism has been spoken of, and also to remind one of the speakers that the saint whom he quoted was the same who said, 'The greatest of these is charity.'

"Whatever defects and imperfections may attach to a few points of the doctrinal system of Calvin—the bulk of which was simply what all Christians believe—it will be found that Calvanism, or *any other ism*, which claims an open Bible and proclaims a crucified and risen Christ, is infinitely preferable to *any form* of polite and polished skepticism, which gathers as its votaries the degenerate sons of heroic ancestors, who, having been trained in a society, and educated in schools, the foundations of which were laid by men of faith and piety, now turn and kick down the ladder by which they have climbed up, and persuade men to live without God and leave them to die without hope.

"The worst kind of religion is no religion at all; and these men, living in ease and luxury, indulging themselves in 'the amusement of going without a religion,' may be thankful that they live in lands where the gospel they neglect has tamed the beastliness and ferocity of the men who, but for Christianity, might long ago have eaten their carcasses like the South Sea Islanders, or cut off their heads and tanned their hides like the monsters of the French Revolution.

"When the microscopic search of skepticism, which has hunted the heavens and sounded the seas to disprove the existence of a creator, has turned its attention to human society, and has found a place on this planet ten miles square where a decent man can live in decency, comfort and security, supporting and educating his children unspoiled and unpolluted; a place where age is reverenced, infancy protected, manhood respected, womanhood honored, and human life held in due regard; when skeptics can find such a place ten miles square on this globe, where the gospel of Christ has not gone, and cleared the way and laid the foundations, and made decency and security possible, it will then be in order for the skeptical *literati* to move thither and then ventilate their views. But so long as these very men are dependent upon the religion which they discard for every privilege they enjoy, they may well hesitate a little before they seek to rob the Christian of his hope, and humanity of its faith in that Saviour who alone has given to man that hope of life eternal which makes life tolerable, and society possible, and robs death of its terrors and the grave of its gloom."—*Our Work at Home.*

